# Supplementary material for: Female responses to experimental removal of sexual selection components in Drosophila melanogaster
Source: BMC Evol Biol. 2014 Nov 19;14:239. doi: 10.1186/s12862-014-0239-3 (PMC4243381; doi:10.1186/s12862-014-0239-3)
Supplement: Additional file 1: — Additional analyses outlining module characteristics, mating frequency data in base population and GO analyses for each module. [file 12862_2014_239_MOESM1_ESM.pdf]

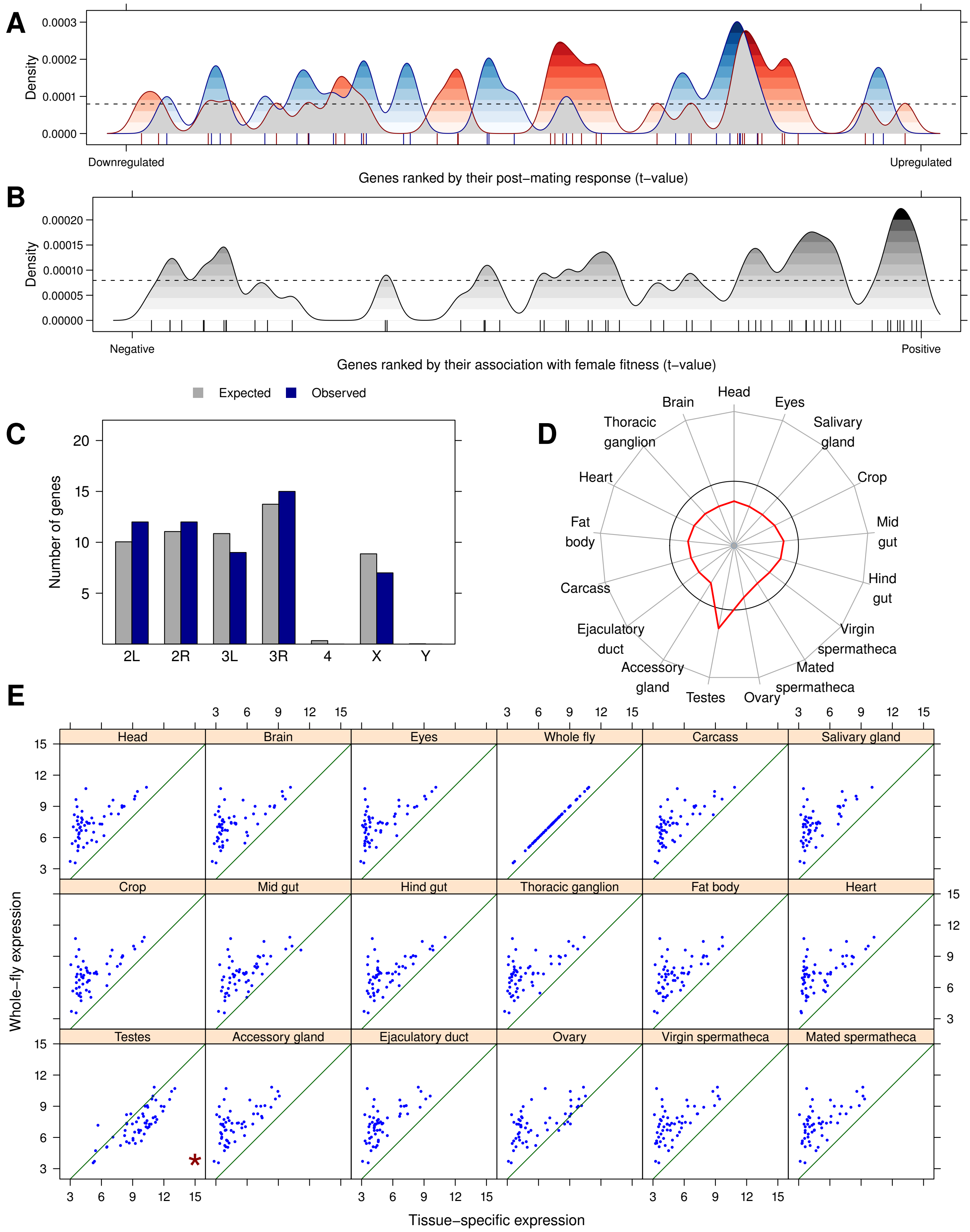

Fig. S1: Module 1. **(A)** Density distribution of significant up-regulated (blue) and down-regulated (red) transcripts along all the tested genes, ranked according to their post-mating reaction (data from a previously published study on the same population; Innocenti and Morrow, 2009); **(B)** Density distribution of the significant transcripts along all the tested genes, ranked by the t-value of their association with female fitness (data from a previously published study on the same population; Innocenti and Morrow, 2010); **(C)** Chromosomal distribution of significant genes ('*' indicates $P < 0.01$ for a Fisher's exact test); **(D)** Average levels of tissue-specificity in expression. The range of the dataset is delimited by the center (minimum) and the external perimeter (maximum). The circular black line indicates the average expression in the whole body. The red line represents the expression in each tissue.; **(E)** Scatterplot of gene expression in different tissues. The green line represents $y = x - 1$ (on log_2 scale, expression in a tissue two-fold the whole fly), while '*' indicates $P < 0.01$ for a Bonferroni-corrected Fisher's exact test with $n = 17$.

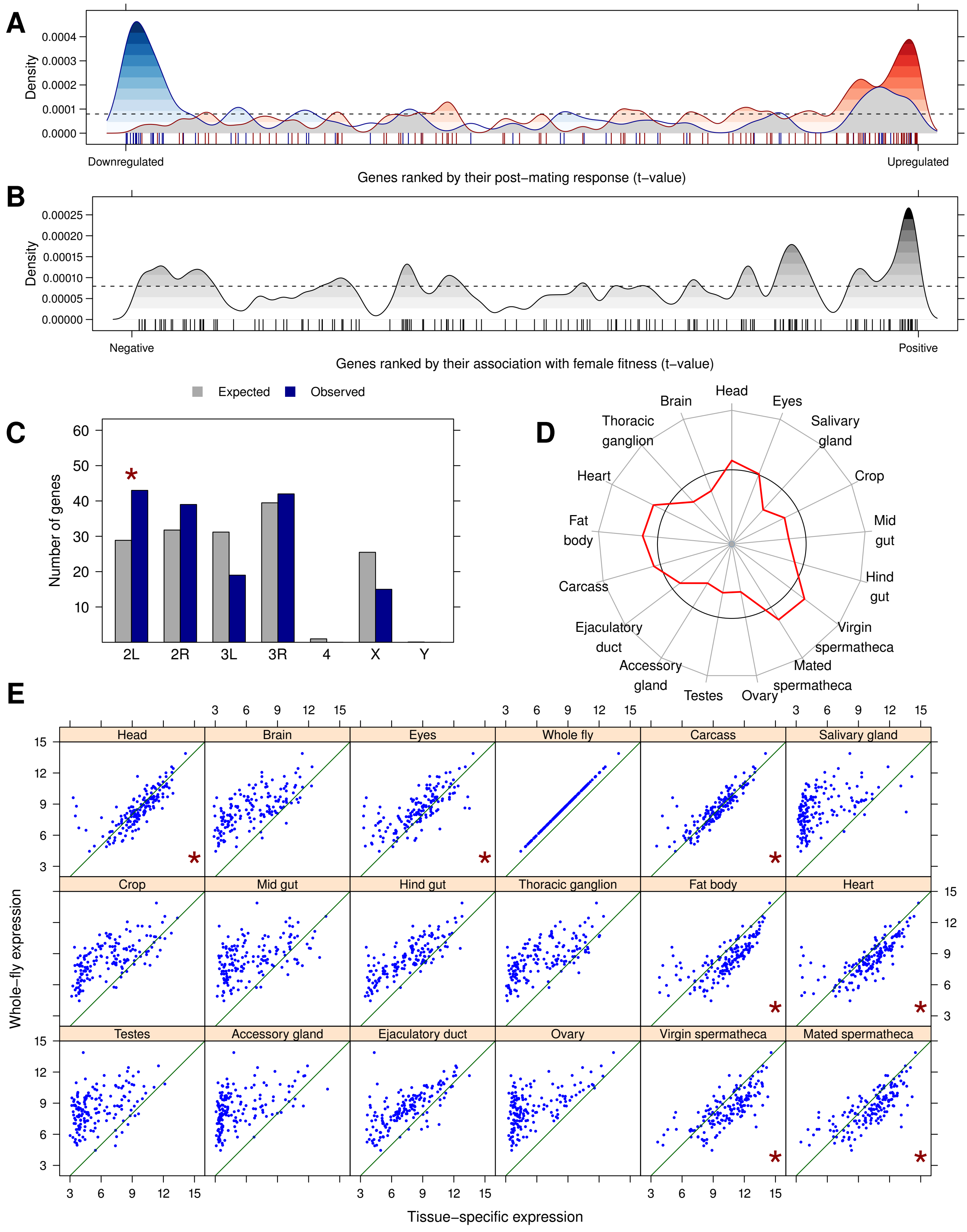

Fig. S2: Module 2. **(A)** Density distribution of significant up-regulated (blue) and down-regulated (red) transcripts along all the tested genes, ranked according to their post-mating reaction (data from a previously published study on the same population; Innocenti and Morrow, 2009); **(B)** Density distribution of the significant transcripts along all the tested genes, ranked by the t-value of their association with female fitness (data from a previously published study on the same population; Innocenti and Morrow, 2010); **(C)** Chromosomal distribution of significant genes ('*' indicates $P < 0.01$ for a Fisher's exact test); **(D)** Average levels of tissue-specificity in expression. The range of the dataset is delimited by the center (minimum) and the external perimeter (maximum). The circular black line indicates the average expression in the whole body. The red line represents the expression in each tissue.; **(E)** Scatterplot of gene expression in different tissues. The green line represents $y = x - 1$ (on log_2 scale, expression in a tissue two-fold the whole fly), while '*' indicates $P < 0.01$ for a Bonferroni-corrected Fisher's exact test with $n = 17$.

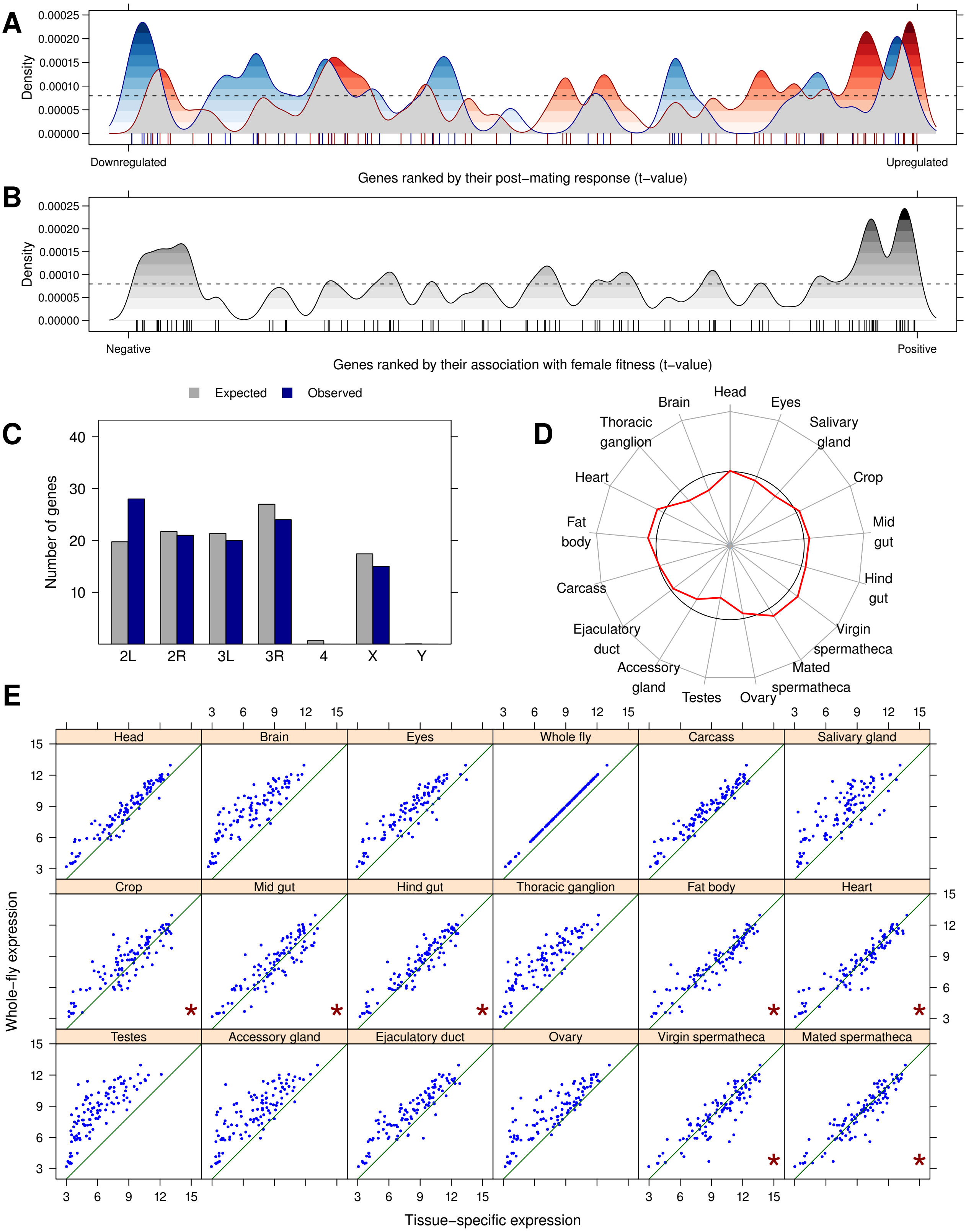

Fig. S3: Module 3. **(A)** Density distribution of significant up-regulated (blue) and down-regulated (red) transcripts along all the tested genes, ranked according to their post-mating reaction (data from a previously published study on the same population; Innocenti and Morrow, 2009); **(B)** Density distribution of the significant transcripts along all the tested genes, ranked by the t-value of their association with female fitness (data from a previously published study on the same population; Innocenti and Morrow, 2010); **(C)** Chromosomal distribution of significant genes ('*' indicates $P < 0.01$ for a Fisher's exact test); **(D)** Average levels of tissue-specificity in expression. The range of the dataset is delimited by the center (minimum) and the external perimeter (maximum). The circular black line indicates the average expression in the whole body. The red line represents the expression in each tissue.; **(E)** Scatterplot of gene expression in different tissues. The green line represents $y = x - 1$ (on log_2 scale, expression in a tissue two-fold the whole fly), while '*' indicates $P < 0.01$ for a Bonferroni-corrected Fisher's exact test with $n = 17$.

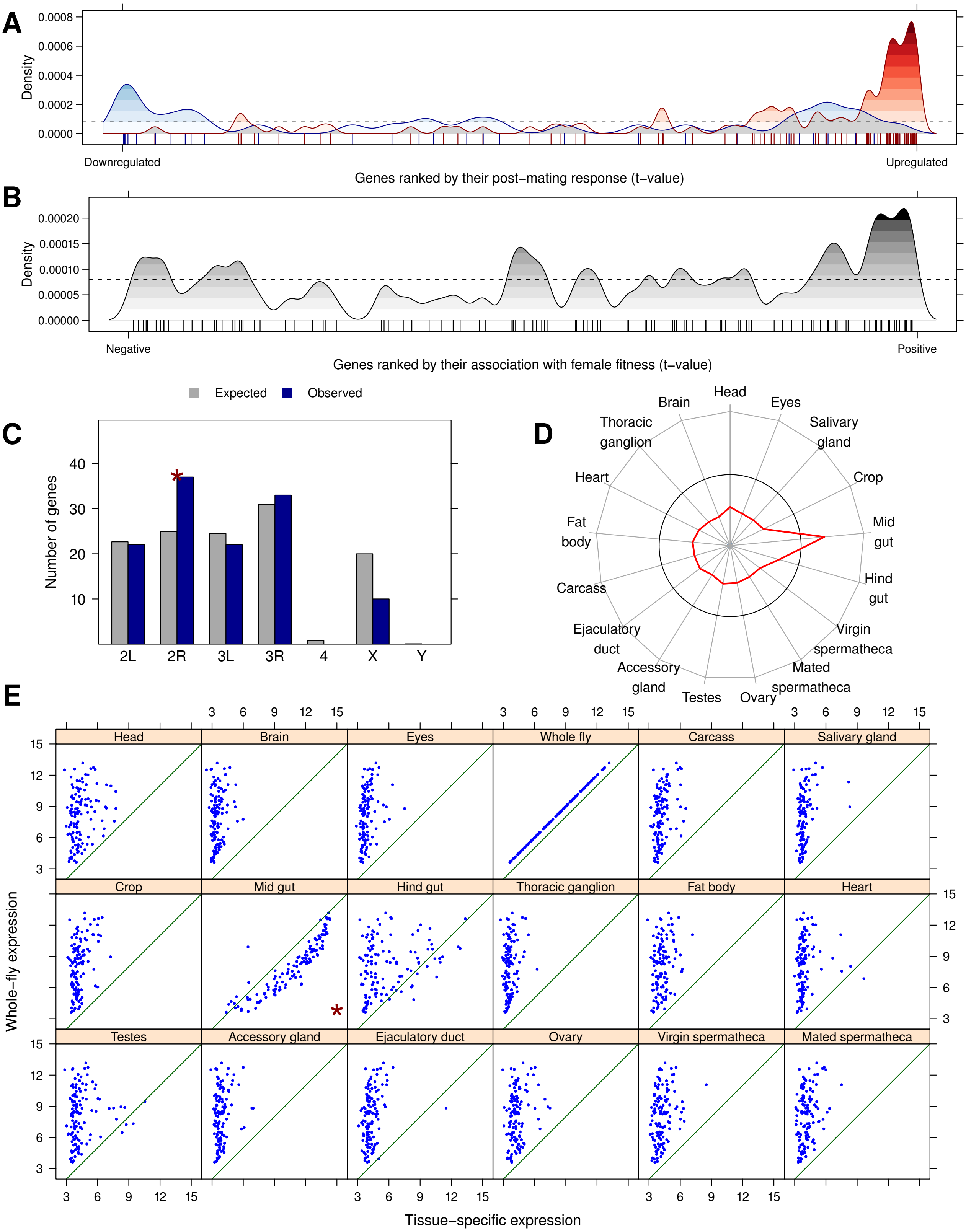

Fig. S4: Module 4. **(A)** Density distribution of significant up-regulated (blue) and down-regulated (red) transcripts along all the tested genes, ranked according to their post-mating reaction (data from a previously published study on the same population; Innocenti and Morrow, 2009); **(B)** Density distribution of the significant transcripts along all the tested genes, ranked by the t-value of their association with female fitness (data from a previously published study on the same population; Innocenti and Morrow, 2010); **(C)** Chromosomal distribution of significant genes ('*' indicates $P < 0.01$ for a Fisher's exact test); **(D)** Average levels of tissue-specificity in expression. The range of the dataset is delimited by the center (minimum) and the external perimeter (maximum). The circular black line indicates the average expression in the whole body. The red line represents the expression in each tissue.; **(E)** Scatterplot of gene expression in different tissues. The green line represents $y = x - 1$ (on log_2 scale, expression in a tissue two-fold the whole fly), while '*' indicates $P < 0.01$ for a Bonferroni-corrected Fisher's exact test with $n = 17$.

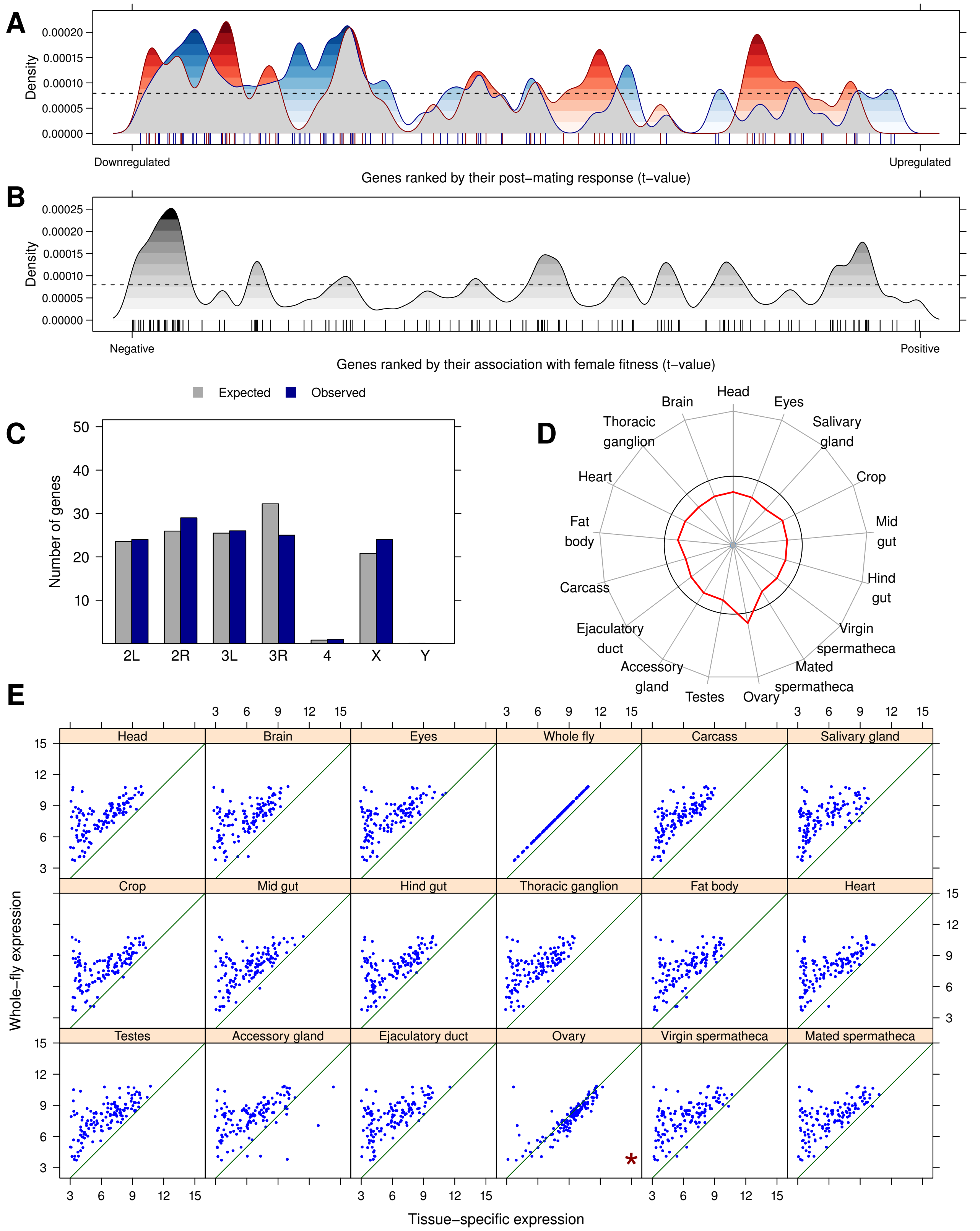

Fig. S5: Module 5. **(A)** Density distribution of significant up-regulated (blue) and down-regulated (red) transcripts along all the tested genes, ranked according to their post-mating reaction (data from a previously published study on the same population; Innocenti and Morrow, 2009); **(B)** Density distribution of the significant transcripts along all the tested genes, ranked by the t-value of their association with female fitness (data from a previously published study on the same population; Innocenti and Morrow, 2010); **(C)** Chromosomal distribution of significant genes ('*' indicates $P < 0.01$ for a Fisher's exact test); **(D)** Average levels of tissue-specificity in expression. The range of the dataset is delimited by the center (minimum) and the external perimeter (maximum). The circular black line indicates the average expression in the whole body. The red line represents the expression in each tissue.; **(E)** Scatterplot of gene expression in different tissues. The green line represents $y = x - 1$ (on log_2 scale, expression in a tissue two-fold the whole fly), while '*' indicates $P < 0.01$ for a Bonferroni-corrected Fisher's exact test with $n = 17$.

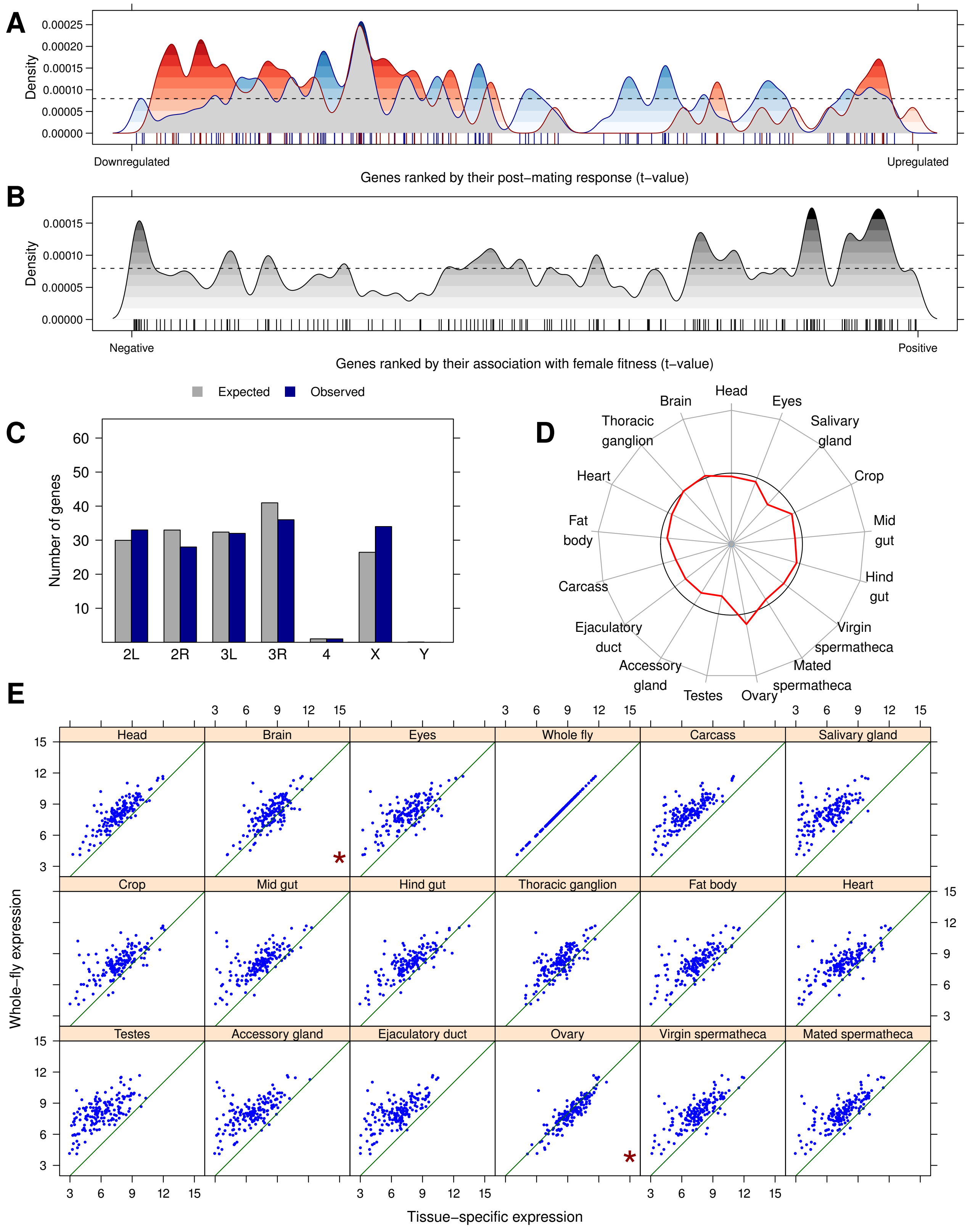

Fig. S6: Module 6. **(A)** Density distribution of significant up-regulated (blue) and down-regulated (red) transcripts along all the tested genes, ranked according to their post-mating reaction (data from a previously published study on the same population; Innocenti and Morrow, 2009); **(B)** Density distribution of the significant transcripts along all the tested genes, ranked by the t-value of their association with female fitness (data from a previously published study on the same population; Innocenti and Morrow, 2010); **(C)** Chromosomal distribution of significant genes ('*' indicates $P < 0.01$ for a Fisher's exact test); **(D)** Average levels of tissue-specificity in expression. The range of the dataset is delimited by the center (minimum) and the external perimeter (maximum). The circular black line indicates the average expression in the whole body. The red line represents the expression in each tissue.; **(E)** Scatterplot of gene expression in different tissues. The green line represents $y = x - 1$ (on log_2 scale, expression in a tissue two-fold the whole fly), while '*' indicates $P < 0.01$ for a Bonferroni-corrected Fisher's exact test with $n = 17$.

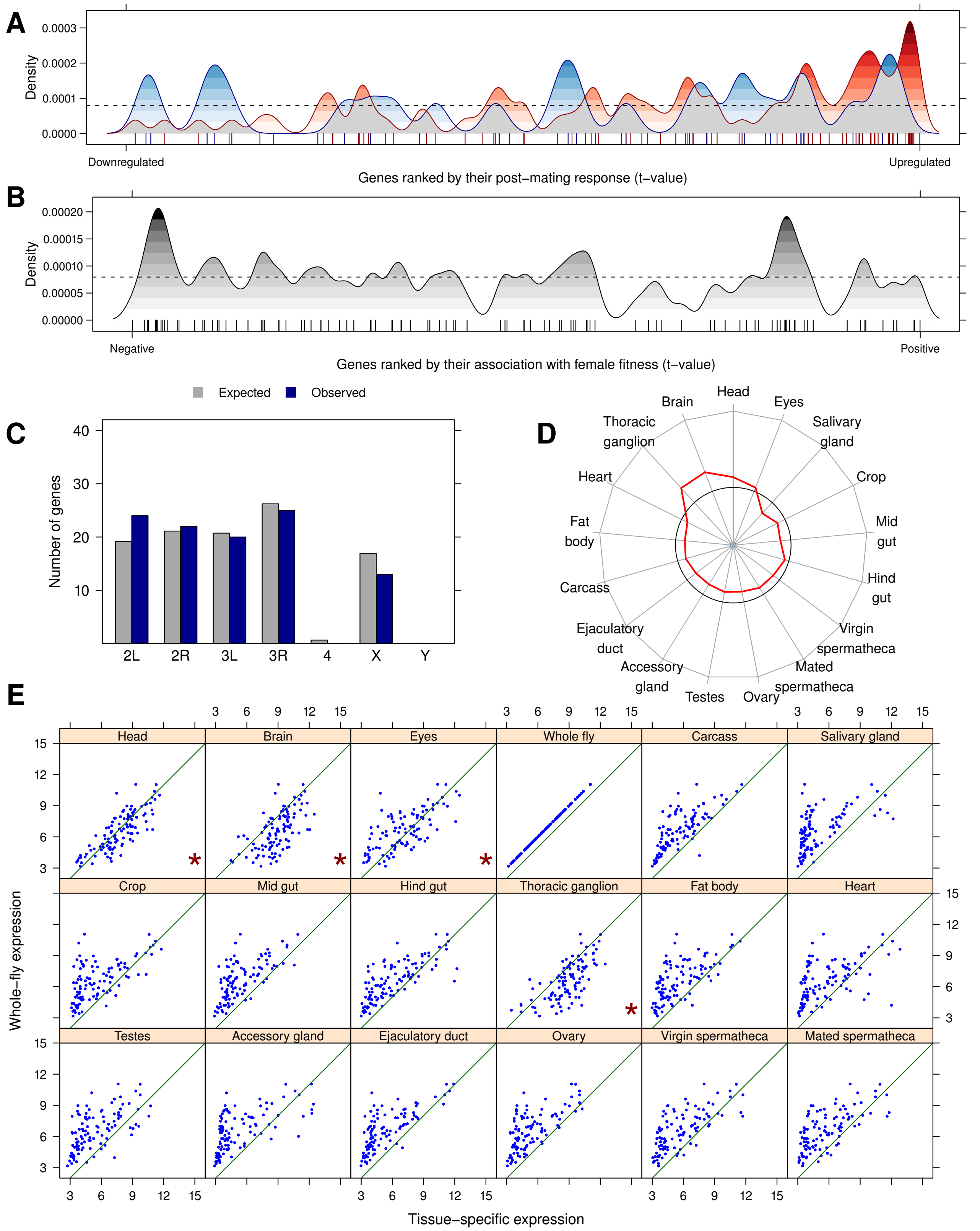

Fig. S7: Module 7. **(A)** Density distribution of significant up-regulated (blue) and down-regulated (red) transcripts along all the tested genes, ranked according to their post-mating reaction (data from a previously published study on the same population; Innocenti and Morrow, 2009); **(B)** Density distribution of the significant transcripts along all the tested genes, ranked by the t-value of their association with female fitness (data from a previously published study on the same population; Innocenti and Morrow, 2010); **(C)** Chromosomal distribution of significant genes ('*' indicates $P < 0.01$ for a Fisher's exact test); **(D)** Average levels of tissue-specificity in expression. The range of the dataset is delimited by the center (minimum) and the external perimeter (maximum). The circular black line indicates the average expression in the whole body. The red line represents the expression in each tissue.; **(E)** Scatterplot of gene expression in different tissues. The green line represents $y = x - 1$ (on log_2 scale, expression in a tissue two-fold the whole fly), while '*' indicates $P < 0.01$ for a Bonferroni-corrected Fisher's exact test with $n = 17$.

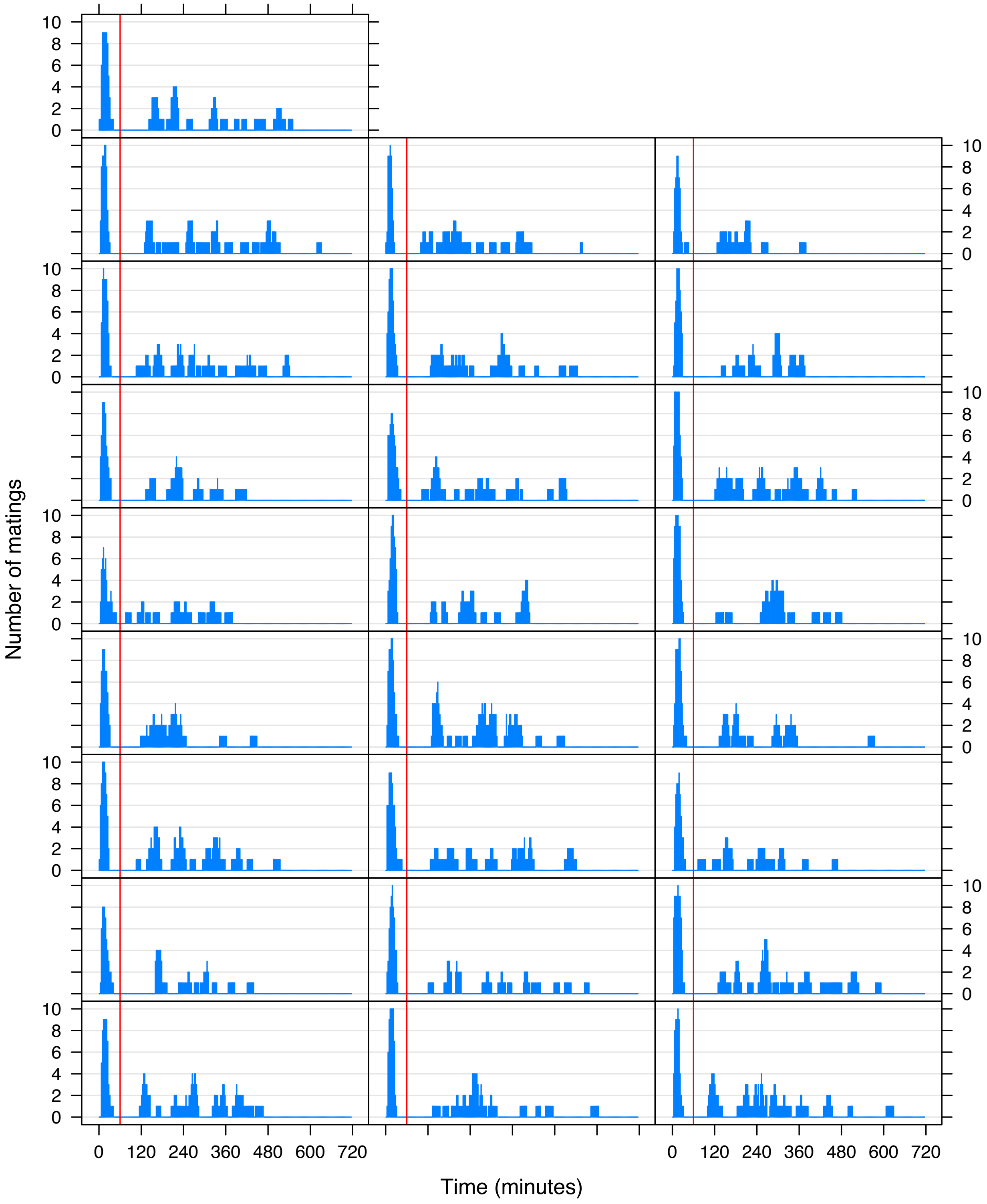

Fig. S8: Mating frequency in the LH$_M$ population. Number of pairs engaged in mating over 12 h in each of the 25 test vials, containing 10 males and 10 females. Data were obtained with time-lapse photography (3 minutes intervals between frames). Vertical red line: 60 minutes.

Table S1: GO terms significantly enriched for genes in module 1 (Hypergeometric test for over-representation, $P < 0.01$)

	GO BP ID	P	Odds	Exp	Obs	Size	GO Term: Biological Process
1	GO:0045333	0.00	13.19	0.36	4	86	cellular respiration
2	GO:0042773	0.00	14.56	0.24	3	57	ATP synthesis coupled electron transport
3	GO:0022900	0.00	13.55	0.25	3	61	electron transport chain
4	GO:0016117	0.00	Inf	0.00	1	1	carotenoid biosynthetic process
5	GO:0030150	0.00	Inf	0.00	1	1	protein import into mitochondrial matrix
6	GO:0070585	0.01	20.34	0.11	2	27	protein localization in mitochondrion
7	GO:0006120	0.01	19.56	0.12	2	28	mitochondrial electron transport, NADH to ubiquinone
8	GO:0006855	0.01	246.84	0.01	1	2	multidrug transport
	**GO MF ID**	**P**	**Odds**	**Exp**	**Obs**	**Size**	**GO Term: Molecular Function**
1	GO:0000774	0.00	Inf	0.00	1	1	adenyl-nucleotide exchange factor activity
2	GO:0003914	0.00	Inf	0.00	1	1	DNA (6-4) photolyase activity
3	GO:0017033	0.00	Inf	0.00	1	1	DNA topoisomerase I binding
4	GO:0003954	0.01	16.59	0.14	2	33	NADH dehydrogenase activity
5	GO:0051287	0.01	14.69	0.15	2	37	NAD or NADH binding
	**GO CC ID**	**P**	**Odds**	**Exp**	**Obs**	**Size**	**GO Term: Cellular Component**
1	GO:0005739	0.00	7.72	1.76	9	465	mitochondrion
2	GO:0031975	0.00	7.84	1.00	6	264	envelope
3	GO:0005740	0.00	8.55	0.73	5	194	mitochondrial envelope
4	GO:0031090	0.00	5.73	1.34	6	354	organelle membrane
5	GO:0070469	0.00	14.67	0.24	3	64	respiratory chain
6	GO:0005743	0.00	8.18	0.59	4	155	mitochondrial inner membrane
7	GO:0005737	0.00	3.40	7.03	14	1859	cytoplasm
8	GO:0005747	0.01	17.88	0.13	2	34	mitochondrial respiratory chain complex I
9	GO:0030964	0.01	17.88	0.13	2	34	NADH dehydrogenase complex

Table S2: GO terms significantly enriched for genes in module 2 (Hypergeometric test for over-representation, $P < 0.01$)

	GO BP ID	P	Odds	Exp	Obs	Size	GO Term: Biological Process
1	GO:0055114	0.00	4.51	5.76	21	443	oxidation reduction
2	GO:0006508	0.00	3.01	8.29	21	637	proteolysis
3	GO:0042811	0.00	154.78	0.04	2	3	pheromone biosynthetic process
4	GO:0032787	0.00	6.48	1.04	6	80	monocarboxylic acid metabolic process
5	GO:0006633	0.00	12.32	0.29	3	22	fatty acid biosynthetic process
6	GO:0042398	0.00	12.32	0.29	3	22	cellular amino acid derivative biosynthetic process
7	GO:0043436	0.00	3.09	3.18	9	244	oxoacid metabolic process
8	GO:0016053	0.00	6.41	0.69	4	53	organic acid biosynthetic process
9	GO:0042445	0.01	7.54	0.44	3	34	hormone metabolic process
	**GO MF ID**	**P**	**Odds**	**Exp**	**Obs**	**Size**	**GO Term: Molecular Function**
1	GO:0030414	0.00	10.94	0.99	9	74	peptidase inhibitor activity
2	GO:0004867	0.00	12.80	0.77	8	57	serine-type endopeptidase inhibitor activity
3	GO:0020037	0.00	7.27	1.77	11	132	heme binding
4	GO:0009055	0.00	6.87	1.87	11	139	electron carrier activity
5	GO:0070011	0.00	3.38	7.31	21	544	peptidase activity, acting on L-amino acid peptides
6	GO:0004497	0.00	7.01	1.48	9	110	monooxygenase activity
7	GO:0008336	0.00	149.33	0.04	2	3	gamma-butyrobetaine dioxygenase activity
8	GO:0017171	0.00	3.21	3.76	11	280	serine hydrolase activity
9	GO:0015926	0.00	16.12	0.23	3	17	glucosidase activity
10	GO:0004252	0.00	3.24	3.37	10	251	serine-type endopeptidase activity
11	GO:0004553	0.00	5.83	0.94	5	72	hydrolase activity, hydrolyzing O-glycosyl compounds
12	GO:0008241	0.00	37.32	0.08	2	6	peptidyl-dipeptidase activity
13	GO:0004222	0.00	5.28	1.04	5	77	metalloendopeptidase activity
14	GO:0003824	0.00	1.93	21.02	32	1912	catalytic activity
15	GO:0005506	0.00	6.36	0.69	4	56	iron ion binding
16	GO:0016787	0.01	1.84	18.23	29	1450	hydrolase activity
17	GO:0004197	0.01	8.67	0.39	3	29	cysteine-type endopeptidase activity
18	GO:0016717	0.01	18.65	0.13	2	10	oxidoreductase activity, acting on paired donors, with oxidation of a pair of donors resulting in the reduction of molecular oxygen to two molecules of water
	**GO CC ID**	**P**	**Odds**	**Exp**	**Obs**	**Size**	**GO Term: Cellular Component**
1	GO:0005792	0.00	17.27	0.77	10	82	microsome
2	GO:0005624	0.00	14.78	0.89	10	94	membrane fraction
3	GO:0000267	0.00	14.10	0.93	10	98	cell fraction
4	GO:0005811	0.00	4.71	2.23	9	236	lipid particle
5	GO:0005576	0.00	3.74	3.84	12	406	extracellular region
6	GO:0005604	0.00	24.07	0.10	2	11	basement membrane
7	GO:0031012	0.01	8.44	0.40	3	42	extracellular matrix

Table S3: GO terms significantly enriched for genes in module 3 (Hypergeometric test for over-representation, $P < 0.01$)

	GO BP ID	P	Odds	Exp	Obs	Size	GO Term: Biological Process
1	GO:0055114	0.00	5.55	4.44	19	443	oxidation reduction
2	GO:0006098	0.00	40.55	0.07	2	7	pentose-phosphate shunt
3	GO:0006739	0.00	40.55	0.07	2	7	NADP metabolic process
4	GO:0019915	0.00	40.55	0.07	2	7	lipid storage
5	GO:0009820	0.00	25.33	0.10	2	10	alkaloid metabolic process
6	GO:0046112	0.00	25.33	0.10	2	10	nucleobase biosynthetic process
7	GO:0019362	0.01	22.52	0.11	2	11	pyridine nucleotide metabolic process
8	GO:0019320	0.01	9.31	0.36	3	36	hexose catabolic process
9	GO:0006090	0.01	18.42	0.13	2	13	pyruvate metabolic process
10	GO:0006725	0.01	5.66	0.77	4	77	cellular aromatic compound metabolic process
11	GO:0044275	0.01	7.87	0.42	3	42	cellular carbohydrate catabolic process
12	GO:0046164	0.01	7.87	0.42	3	42	alcohol catabolic process
13	GO:0005996	0.01	5.22	0.83	4	83	monosaccharide metabolic process
14	GO:0046395	0.01	7.48	0.44	3	44	carboxylic acid catabolic process
15	GO:0043603	0.01	15.58	0.15	2	15	cellular amide metabolic process
	GO MF ID	P	Odds	Exp	Obs	Size	GO Term: Molecular Function
1	GO:0016491	0.00	6.40	4.59	22	461	oxidoreductase activity
2	GO:0008374	0.00	24.56	0.21	4	20	O-acyltransferase activity
3	GO:0009055	0.00	6.21	1.46	8	139	electron carrier activity
4	GO:0004095	0.00	Inf	0.02	2	2	carnitine O-palmitoyltransferase activity
5	GO:0017159	0.00	192.32	0.03	2	3	pantetheine hydrolase activity
6	GO:0048037	0.00	5.03	1.78	8	169	cofactor binding
7	GO:0003995	0.00	24.28	0.16	3	15	acyl-CoA dehydrogenase activity
8	GO:0004364	0.00	12.25	0.38	4	36	glutathione transferase activity
9	GO:0016616	0.00	8.30	0.68	5	66	oxidoreductase activity, acting on the CH-OH group of donors, NAD or NADP as acceptor
10	GO:0004616	0.00	96.15	0.04	2	4	phosphogluconate dehydrogenase (decarboxylating) activity
11	GO:0016747	0.00	5.80	1.15	6	109	transferase activity, transferring acyl groups other than amino-acyl groups
12	GO:0004497	0.00	5.74	1.16	6	110	monooxygenase activity
13	GO:0016409	0.00	64.09	0.05	2	5	palmitoyltransferase activity
14	GO:0020037	0.00	4.73	1.39	6	132	heme binding
15	GO:0050660	0.00	7.24	0.61	4	58	FAD binding
16	GO:0032934	0.00	24.02	0.11	2	10	sterol binding
17	GO:0003824	0.01	1.95	23.85	34	3071	catalytic activity
	GO CC ID	P	Odds	Exp	Obs	Size	GO Term: Cellular Component
1	GO:0005811	0.00	6.59	2.08	11	236	lipid particle
2	GO:0005737	0.00	3.26	16.39	32	1859	cytoplasm
3	GO:0005739	0.00	7.98	1.05	7	132	mitochondrion
4	GO:0005792	0.01	6.13	0.72	4	82	microsome
5	GO:0044429	0.01	3.06	2.94	8	333	mitochondrial part
6	GO:0009368	0.01	Inf	0.01	1	1	endopeptidase Clp complex
7	GO:0019898	0.01	7.69	0.43	3	49	extrinsic to membrane
8	GO:0005624	0.01	5.30	0.83	4	94	membrane fraction

Supporting information

Table S4: GO terms significantly enriched for genes in module 4 (Hypergeometric test for over-representation, $P < 0.01$)

	GO BP ID	P	Odds	Exp	Obs	Size	GO Term: Biological Process
1	GO:0006508	0.00	5.95	6.80	28	637	proteolysis
2	GO:0005975	0.00	5.41	2.15	10	217	carbohydrate metabolic process
3	GO:0005976	0.00	6.02	1.31	7	123	polysaccharide metabolic process
4	GO:0006030	0.00	6.98	0.97	6	91	chitin metabolic process
5	GO:0008152	0.00	2.21	45.45	60	4260	metabolic process
6	GO:0005996	0.00	6.26	0.89	5	83	monosaccharide metabolic process
7	GO:0009166	0.00	27.13	0.10	2	9	nucleotide catabolic process
8	GO:0006013	0.00	23.73	0.11	2	10	mannose metabolic process
	GO MF ID	P	Odds	Exp	Obs	Size	GO Term: Molecular Function
1	GO:0017171	0.00	8.53	2.91	19	280	serine hydrolase activity
2	GO:0004252	0.00	8.30	2.61	17	251	serine-type endopeptidase activity
3	GO:0070011	0.00	7.24	3.45	19	361	peptidase activity, acting on L-amino acid peptides
4	GO:0004558	0.00	62.92	0.14	5	13	alpha-glucosidase activity
5	GO:0016787	0.00	3.58	12.27	30	1450	hydrolase activity
6	GO:0004553	0.00	10.22	0.93	8	89	hydrolase activity, hydrolyzing O-glycosyl compounds
7	GO:0030246	0.00	6.74	1.54	9	148	carbohydrate binding
8	GO:0008238	0.00	8.22	0.98	7	94	exopeptidase activity
9	GO:0001871	0.00	7.60	1.05	7	101	pattern binding
10	GO:0004181	0.00	18.92	0.26	4	25	metallocarboxypeptidase activity
11	GO:0008061	0.00	8.55	0.80	6	77	chitin binding
12	GO:0008237	0.00	5.59	1.61	8	155	metallopeptidase activity
13	GO:0004806	0.00	12.40	0.37	4	36	triglyceride lipase activity
14	GO:0008253	0.00	64.84	0.05	2	5	5'-nucleotidase activity
15	GO:0016811	0.00	7.47	0.59	4	57	hydrolase activity, acting on carbon-nitrogen (but not peptide) bonds, in linear amides
16	GO:0004559	0.00	27.77	0.09	2	9	alpha-mannosidase activity
17	GO:0008970	0.00	27.77	0.09	2	9	phospholipase A1 activity
18	GO:0015103	0.01	8.65	0.39	3	37	inorganic anion transmembrane transporter activity
	GO CC ID	P	Odds	Exp	Obs	Size	GO Term: Cellular Component
1	GO:0005764	0.00	42.76	0.13	4	23	lysosome
2	GO:0005773	0.00	16.85	0.29	4	52	vacuole
3	GO:0005576	0.00	5.16	2.24	9	406	extracellular region
4	GO:0005615	0.00	11.52	0.30	3	54	extracellular space

Table S5: GO terms significantly enriched for genes in module 5 (Hypergeometric test for over-representation, $P < 0.01$)

	GO BP ID	P	Odds	Exp	Obs	Size	GO Term: Biological Process
1	GO:0007292	0.00	3.96	3.85	13	384	female gamete generation
2	GO:0033261	0.00	34.23	0.12	3	12	regulation of S phase
3	GO:0051325	0.00	12.58	0.37	4	37	interphase
4	GO:0019953	0.00	3.17	5.13	14	512	sexual reproduction
5	GO:0045035	0.00	101.41	0.04	2	4	sensory organ precursor cell division
6	GO:0010605	0.00	4.23	2.40	9	240	negative regulation of macromolecule metabolic process
7	GO:0007281	0.00	4.63	1.94	8	194	germ cell development
8	GO:0048609	0.00	2.97	5.44	14	543	reproductive process in a multicellular organism
9	GO:0033554	0.00	4.63	1.68	7	168	cellular response to stress
10	GO:0031400	0.00	50.69	0.06	2	6	negative regulation of protein modification process
11	GO:0045749	0.00	50.69	0.06	2	6	negative regulation of S phase of mitotic cell cycle
12	GO:0048132	0.00	50.69	0.06	2	6	female germ-line stem cell division
13	GO:0006541	0.00	40.55	0.07	2	7	glutamine metabolic process
14	GO:0032268	0.00	5.16	1.06	5	106	regulation of cellular protein metabolic process
15	GO:0048646	0.00	3.15	3.17	9	316	anatomical structure formation involved in morphogenesis
16	GO:0048519	0.00	2.66	5.07	12	506	negative regulation of biological process
17	GO:0031324	0.00	3.65	2.10	7	210	negative regulation of cellular metabolic process
18	GO:0009994	0.00	4.91	1.11	5	111	oocyte differentiation
19	GO:0031047	0.01	9.03	0.37	3	37	gene silencing by RNA
20	GO:0000082	0.01	20.26	0.12	2	12	G1/S transition of mitotic cell cycle
21	GO:0042770	0.01	20.26	0.12	2	12	DNA damage response, signal transduction
22	GO:0006139	0.01	1.94	13.94	23	1391	nucleobase, nucleoside, nucleotide and nucleic acid metabolic process
23	GO:0001709	0.01	4.37	1.24	5	124	cell fate determination
24	GO:0006281	0.01	5.43	0.80	4	80	DNA repair
25	GO:0006378	0.01	16.88	0.14	2	14	mRNA polyadenylation
	**GO MF ID**	**P**	**Odds**	**Exp**	**Obs**	**Size**	**GO Term: Molecular Function**
1	GO:0005528	0.00	47.52	0.06	2	6	FK506 binding
2	GO:0003676	0.00	1.97	14.78	25	1389	nucleic acid binding
3	GO:0016884	0.01	18.99	0.13	2	12	carbon-nitrogen ligase activity, with glutamine as amido-N-donor
	**GO CC ID**	**P**	**Odds**	**Exp**	**Obs**	**Size**	**GO Term: Cellular Component**
1	GO:0043186	0.01	20.50	0.12	2	11	P granule

Table S6: GO terms significantly enriched for genes in module 6 (Hypergeometric test for over-representation, $P < 0.01$)

	GO BP ID	P	Odds	Exp	Obs	Size	GO Term: Biological Process
1	GO:0023046	0.00	2.93	12.09	29	801	signaling process
2	GO:0050789	0.00	2.19	29.30	49	1941	regulation of biological process
3	GO:0016081	0.00	16.86	0.30	4	20	synaptic vesicle docking during exocytosis
4	GO:0016080	0.00	33.46	0.14	3	9	synaptic vesicle targeting
5	GO:0051301	0.00	4.63	2.17	9	144	cell division
6	GO:0051650	0.00	25.09	0.17	3	11	establishment of vesicle localization
7	GO:0007154	0.00	2.75	6.97	17	462	cell communication
8	GO:0007610	0.00	3.06	5.12	14	339	behavior
9	GO:0048278	0.00	12.25	0.39	4	26	vesicle docking
10	GO:0042078	0.00	10.78	0.44	4	29	germ-line stem cell division
11	GO:0006979	0.00	7.53	0.75	5	50	response to oxidative stress
12	GO:0007163	0.00	4.75	1.63	7	108	establishment or maintenance of cell polarity
13	GO:0048489	0.00	5.31	1.25	6	83	synaptic vesicle transport
14	GO:0009888	0.00	2.62	6.35	15	421	tissue development
15	GO:0019953	0.00	2.45	7.73	17	512	sexual reproduction
16	GO:0009653	0.00	2.50	7.21	16	520	anatomical structure morphogenesis
17	GO:0007242	0.00	2.75	5.21	13	345	intracellular signaling cascade
18	GO:0048609	0.00	2.30	8.20	17	543	reproductive process in a multicellular organism
19	GO:0032989	0.00	2.29	8.21	17	544	cellular component morphogenesis
20	GO:0007362	0.00	11.79	0.30	3	20	terminal region determination
21	GO:0046622	0.00	33.18	0.09	2	6	positive regulation of organ growth
22	GO:0048732	0.00	3.52	2.48	8	164	gland development
23	GO:0002009	0.00	3.21	3.05	9	202	morphogenesis of an epithelium
24	GO:0007350	0.00	3.39	2.57	8	170	blastoderm segmentation
25	GO:0000087	0.00	3.73	2.04	7	135	M phase of mitotic cell cycle
26	GO:0000288	0.00	26.54	0.11	2	7	nuclear-transcribed mRNA catabolic process, deadenylation-dependent decay
27	GO:0046854	0.00	26.54	0.11	2	7	phosphoinositide phosphorylation
28	GO:0007292	0.01	2.45	5.80	13	384	female gamete generation
29	GO:0007399	0.01	2.13	9.34	18	619	nervous system development
30	GO:0065008	0.01	2.17	8.62	17	571	regulation of biological quality
31	GO:0035272	0.01	3.59	2.11	7	140	exocrine system development
32	GO:0023034	0.01	2.49	5.24	12	347	intracellular signaling pathway
33	GO:0016318	0.01	9.11	0.38	3	25	ommatidial rotation
34	GO:0008595	0.01	3.92	1.66	6	110	anterior/posterior axis specification, embryo
35	GO:0009790	0.01	2.28	6.70	14	444	embryonic development
36	GO:0003002	0.01	2.41	5.40	12	358	regionalization
37	GO:0033206	0.01	18.95	0.14	2	9	cytokinesis after meiosis
38	GO:0007435	0.01	3.73	1.74	6	115	salivary gland morphogenesis
39	GO:0007165	0.01	2.63	4.13	10	294	signal transduction
40	GO:0048663	0.01	5.48	0.80	4	53	neuron fate commitment
41	GO:0007275	0.01	1.85	14.68	24	1042	multicellular organismal development
42	GO:0001745	0.01	2.92	2.94	8	195	compound eye morphogenesis
43	GO:0017148	0.01	7.70	0.44	3	29	negative regulation of translation
44	GO:0048468	0.01	1.96	10.69	19	708	cell development
	**GO MF ID**	**P**	**Odds**	**Exp**	**Obs**	**Size**	**GO Term: Molecular Function**
1	GO:0005515	0.00	2.10	24.60	42	1697	protein binding
2	GO:0016303	0.00	138.16	0.04	2	3	1-phosphatidylinositol-3-kinase activity
3	GO:0035005	0.00	138.16	0.04	2	3	phosphatidylinositol-4-phosphate 3-kinase activity
4	GO:0046934	0.00	138.16	0.04	2	3	phosphatidylinositol-4,5-bisphosphate 3-kinase activity
5	GO:0008374	0.00	12.27	0.29	3	20	O-acyltransferase activity
6	GO:0001727	0.01	23.01	0.12	2	8	lipid kinase activity
7	GO:0005057	0.01	5.37	0.81	4	56	receptor signaling protein activity
	**GO CC ID**	**P**	**Odds**	**Exp**	**Obs**	**Size**	**GO Term: Cellular Component**
1	GO:0005942	0.00	67.25	0.06	2	4	phosphoinositide 3-kinase complex
2	GO:0005881	0.00	33.61	0.09	2	6	cytoplasmic microtubule
3	GO:0005622	0.01	1.77	55.91	68	3738	intracellular
4	GO:0005763	0.01	8.13	0.42	3	28	mitochondrial small ribosomal subunit

Table S7: GO terms significantly enriched for genes in module 7 (Hypergeometric test for over-representation, $P < 0.01$)

	GO BP ID	P	Odds	Exp	Obs	Size	GO Term: Biological Process
1	GO:0055085	0.00	3.64	3.82	12	408	transmembrane transport
2	GO:0051239	0.00	3.86	2.63	9	281	regulation of multicellular organismal process
3	GO:0006885	0.00	54.35	0.06	2	6	regulation of pH
4	GO:0042332	0.00	43.47	0.07	2	7	gravitaxis
5	GO:0016202	0.00	12.69	0.27	3	29	regulation of striated muscle tissue development
6	GO:0051234	0.00	2.17	12.89	23	1376	establishment of localization
7	GO:0048878	0.00	7.16	0.62	4	66	chemical homeostasis
8	GO:0055080	0.00	10.99	0.31	3	33	cation homeostasis
9	GO:0047484	0.01	Inf	0.01	1	1	regulation of response to osmotic stress
10	GO:0048491	0.01	Inf	0.01	1	1	retrograde synaptic vesicle transport
	GO MF ID	P	Odds	Exp	Obs	Size	GO Term: Molecular Function
1	GO:0022891	0.00	3.48	5.43	16	560	substrate-specific transmembrane transporter activity
2	GO:0015291	0.00	9.38	0.74	6	80	secondary active transmembrane transporter activity
3	GO:0015491	0.00	45.34	0.10	3	10	cation:cation antiporter activity
4	GO:0005215	0.00	3.04	7.50	19	773	transporter activity
5	GO:0008324	0.00	3.97	3.47	12	358	cation transmembrane transporter activity
6	GO:0015385	0.00	104.54	0.04	2	4	sodium:hydrogen antiporter activity
7	GO:0015293	0.00	7.58	0.74	5	76	symporter activity
8	GO:0008440	0.00	69.69	0.05	2	5	inositol trisphosphate 3-kinase activity
9	GO:0015300	0.00	12.67	0.27	3	28	solute:solute antiporter activity
10	GO:0015370	0.00	7.75	0.57	4	59	solute:sodium symporter activity
11	GO:0015299	0.00	23.21	0.11	2	11	solute:hydrogen antiporter activity
12	GO:0004428	0.01	17.40	0.14	2	14	inositol or phosphatidylinositol kinase activity
13	GO:0003867	0.01	Inf	0.01	1	1	4-aminobutyrate transaminase activity
14	GO:0005034	0.01	Inf	0.01	1	1	osmosensor activity
15	GO:0005219	0.01	Inf	0.01	1	1	ryanodine-sensitive calcium-release channel activity
16	GO:0008432	0.01	Inf	0.01	1	1	JUN kinase binding
17	GO:0008506	0.01	Inf	0.01	1	1	sucrose:hydrogen symporter activity
18	GO:0008805	0.01	Inf	0.01	1	1	carbon-monoxide oxygenase activity
19	GO:0015154	0.01	Inf	0.01	1	1	disaccharide transmembrane transporter activity
20	GO:0016250	0.01	Inf	0.01	1	1	N-sulfoglucosamine sulfohydrolase activity
21	GO:0016594	0.01	Inf	0.01	1	1	glycine binding
22	GO:0016596	0.01	Inf	0.01	1	1	thienylcyclohexylpiperidine binding
23	GO:0035049	0.01	Inf	0.01	1	1	juvenile hormone acid methyltransferase activity
	GO CC ID	P	Odds	Exp	Obs	Size	GO Term: Cellular Component
1	GO:0016021	0.00	2.95	7.85	18	890	integral to membrane
2	GO:0044425	0.00	2.71	10.88	22	1234	membrane part
3	GO:0005886	0.00	3.29	4.79	13	543	plasma membrane
4	GO:0009925	0.01	Inf	0.01	1	1	basal plasma membrane
